# The Microbiome of Fertilization-Stage Maize Silks (Style) Encodes Genes and Expresses Traits That Potentially Promote Survival in Pollen/Style Niches and Host Reproduction

**DOI:** 10.3390/microorganisms12071473

**Published:** 2024-07-19

**Authors:** Michelle E. H. Thompson, Manish N. Raizada

**Affiliations:** Department of Plant Agriculture, University of Guelph, Guelph, ON N1G 2W1, Canada; michelleehthompson@gmail.com

**Keywords:** style, fertilization, reproduction, bacteria, pollen tube, vertical transmission, abiotic stress, osmoprotectant, gene mining

## Abstract

Within flowers, the style channel receives pollen and transmits male gametes inside elongating pollen tubes to ovules. The styles of maize/corn are called silks. Fertilization-stage silks possess complex microbiomes, which may partially derive from pollen. These microbiomes lack functional analysis. We hypothesize that fertilization-stage silk microbiomes promote host fertilization to ensure their own vertical transmission. We further hypothesize that these microbes encode traits to survive stresses within the silk (water/nitrogen limitation) and pollen (dehydration/aluminum) habitats. Here, bacteria cultured from fertilization-stage silks of 14 North American maize genotypes underwent genome mining and functional testing, which revealed osmoprotection, nitrogen-fixation, and aluminum-tolerance traits. Bacteria contained auxin biosynthesis genes, and testing confirmed indole compound secretion, which is relevant, since pollen delivers auxin to silks to stimulate egg cell maturation. Some isolates encoded biosynthetic/transport compounds known to regulate pollen tube guidance/growth. The isolates encoded ACC deaminase, which degrades the precursor for ethylene that otherwise accelerates silk senescence. The findings suggest that members of the microbiome of fertilization-stage silks encode adaptations to survive the stress conditions of silk/pollen and have the potential to express signaling compounds known to impact reproduction. Overall, whereas these microbial traits have traditionally been assumed to primarily promote vegetative plant growth, this study proposes they may also play selfish roles during host reproduction.

## 1. Introduction

The style tissue of angiosperms is a reproductive channel comparable to the uterus and fallopian tubes in humans, through which male gametes are transmitted to an ovule [1]. In the global staple crop, maize (*Zea mays* L. ssp. *mays*, corn), the styles are the uniquely long, fast-growing tissues referred to as silks: the threads that emerge from the tips of maize cobs [2,3]. Maize is monoecious and wind pollinated (anemophilous); pollen grains are released from the tassel (male inflorescence) and dispersed by wind, with some subsequently landing on a receptive silk that, by necessity, is exposed to the external environment [2]. When a pollen grain germinates on a silk, a pollen tube grows through the long silk channel, through which sperm nuclei migrate [2]. Each silk leads to an ovule, so each of the hundreds of grains of maize on a cob represents the successful transmission of sperm nuclei through a silk, followed by double fertilization and seed formation.

Silks at the critical fertilization stage have been termed “transmitting silks”, as they contain the pollen tube actively transmitting the male gametes to the ovule [4]. Using next generation sequencing (NGS), our group recently showed that transmitting silks have a complex microbiome, termed the transmitting silk microbiome (TSM): >1300 bacterial genera and >5000 taxa were identified in silks of a North American modern maize diversity panel grown in Ontario, Canada [4]. Systematic culturing of the style tissue was first reported using silks from the same field trial [5]. This was the first systematic culture-based analysis from a plant style microbiome to the best of our knowledge. The study also improved the taxonomic resolution of the TSM (from both healthy and *Fusarium graminearum*-treated silks) [5]. However, the functions of these microbes remain unknown, and exploring their potential functions may improve plant production, breeding, targeted treatments, and more generally, our understanding of angiosperm reproduction and plant–microbe interactions. Microbes have also been cultured from exposed silk tissue of randomly selected cobs from select maize fields in Brazil [6].

Maize seeds also have a microbiome [7,8,9]. Hybrid maize seed has been shown to share part of their seed microbiome with both parents [10]. There is evidence of vertical transmission of the plant microbiome [11], with convincing evidence that at least one *Bacillus* taxon is inherited from maize pollen [12]. For bacteria to be vertically transmitted, they would need to be associated with the maternal tissue, or on the paternal side, a successful pollen tube [13,14] and/or male gametes [15]. We hypothesize that the TSM may encode traits that promote host reproductive success (e.g., promote pollen tube growth), thereby ensuring their own long-term survival. Bacteria promote successful mating in some animals [16,17], but it is unknown whether this occurs in plants.

Members of the TSM may originate from the environment, pollen, or from the maternal host plant. The microbes may be vertically transmitted, or, alternatively, they may simply use the nutrient-rich reproductive tissues as habitats, or in the case of maize pollen, as dispersal mechanisms. Regardless, these microbes face abiotic stressors: pollen undergoes dehydration [18] and is rich in toxic aluminum [19]; there are dynamic pH changes in the style tissue which correlates with pollen tube growth [20,21,22]; furthermore, silks can experience water and nitrogen limitations [23,24,25,26]. Additionally, maize reproduction and grain yield are threatened by climate change, largely due to drought [4,27]. Therefore, our second hypothesis is that the TSM encodes traits that promote tolerance to the abiotic stresses of reproductive tissues.

As already noted, fertilization-stage silks from the same field trial were used for NGS [4] and culturing [5]. Here, we selected a subset of the previously reported bacterial isolates from healthy silks [5], focusing on prevalent taxa, and undertook genome mining and functional testing in vitro to test whether the bacteria of fertilization-stage silks show evidence, first, of adaptation to abiotic stressors relevant in pollen and transmitting silks, and second, of signaling molecules known to promote maize reproduction, including auxin, ethylene from its precursor 1-aminocyclopropane-1-carboxylic acid (ACC), gamma-aminobutyric acid (GABA), and nitric oxide.

## 2. Materials and Methods

### 2.1. Methodology Overview

**Figure 1:**
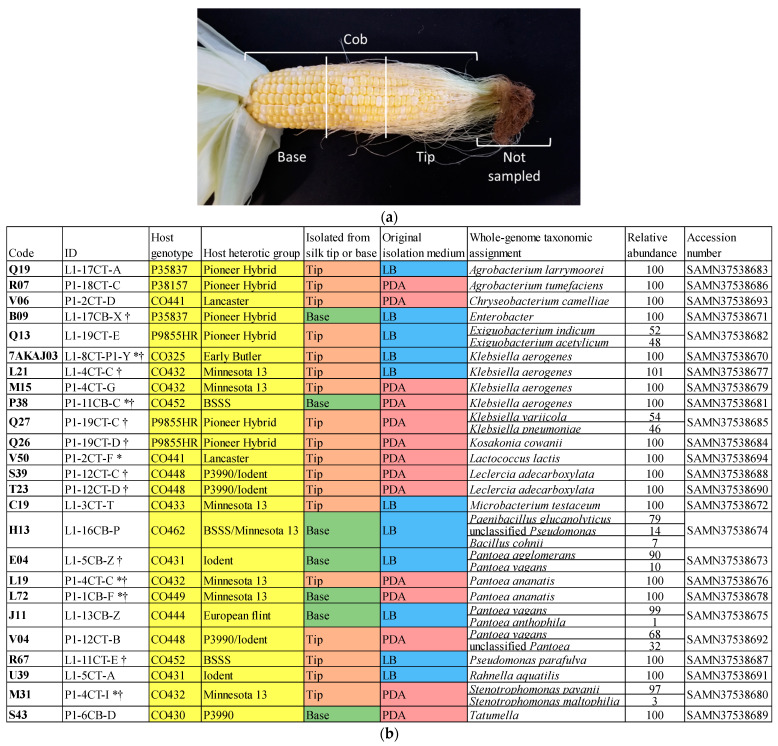
(**a**) Fertilization-stage silk samples were taken from the tip and the base of each cob within the portion covered by husk leaves. See Thompson et al., 2023, Supplemental Materials, Figure S1c [5]. (**b**) Details for bacterial isolates used in this study, including isolate origin (maize host genotype and heterotic group), sample location (tip or base portion of silks), medium that was used to isolate the bacteria from silks (LB agar or potato dextrose agar [PDA]), whole-genome sequencing results (including relative abundance for the assignment of taxonomy), and NCBI GenBank accession number. The asterisks (*) indicate bacterial strains that were previously predicted to be part of the cultured core TSM based on 16S sequences from the cultured library of 748 bacteria. The cultured core was defined as cultured OTUs that were prevalent in both healthy and *F. graminearum*-treated silks, meaning they were cultured from at least three host genotypes in each treatment [5,28]. The obelisks (†) indicate strains that were previously predicted to be part of the core TSM via the matching of 16S sequences to V4-MiSeq-defined core OTUs [4,5,28].

Bacteria were previously cultured from field-grown, open-pollinated silks of 14 maize genotypes that spanned the heterotic groups underlying North American commercial maize germplasm, as described in [5,28]. Briefly, under sterile conditions, the husk-free, exposed ends of the silks were discarded, and the husk-covered silks were separated into segments (Figure 1a). The tip and base portions were then ground and cultured on potato dextrose agar (PDA) and LB agar, and unique individual colonies underwent subsequent culturing, storage, and DNA isolation [5,28]. The microbes were then assigned taxonomic predictions using 16S rDNA sequencing, contig assembly, and BLAST searching, as previously described [5,28]. Here, select bacteria from non-infected (healthy) pollinated silks (focusing on prevalent taxa) underwent in vitro testing, whole-genome sequencing, and gene mining.

### 2.2. In Vitro Testing of Microbial Traits

A subset of bacteria from healthy silk tissues were tested in various assays (Supplementary Text S1). The indole-containing compound production assay (proxy for indoleacetic acid, IAA) was modified from Johnston-Monje and Raizada [7], which was based on Bric et al. [29]. The bacteria were tested for their ability to grow in an anaerobic chamber (Baker-Ruskinn Concept 500, The Baker Company, Inc., Sanford, FL, USA, gas mix: Linde, 10% H_2_, 10% CO_2_, and 80% N_2_) on media without added nitrogen, as adapted from the American Type Culture Collection’s “ATCC medium: 1312 *Azospirillum amazonense* (LGI medium)”, and contained molybdenum to facilitate biological nitrogen fixation. The poly(ethylene glycol) tolerance protocol, a proxy for drought tolerance, was adapted from Hernández-Fernández et al. [30] and Latif et al. [31]. The acid and aluminum tolerance protocol was adapted from Huang et al. [32] and Lim et al. [33].

### 2.3. Whole-Genome Sequencing, Gene Annotation, and Mining

Single-colony liquid cultures of 25 isolates from healthy silk tissues (including representatives from the most prevalent species and OTUs cultured and some special inclusions) were used for bacterial genomic DNA isolation using the DNeasy UltraClean microbial kit (product number 1222450; Qiagen, Hilden, Germany) (Figure 1b). Illumina sequencing was conducted by third parties (Lavie Bio Ltd., Rehovot, Israel, and MiGS, Pittsburgh, PA, USA). Both Lavie Bio and MiGS sequences were bioinformatically analyzed at MiGS. The gene annotation files for the isolates were combined into one Excel file to search for genes of interest. Further details can be found in Supplementary Text S2.

## 3. Results

### 3.1. Overview of Whole-Genome Mining and In Vitro Testing for Traits Relevant to the Silk Environment

To test for microbial traits relevant for promoting silk health and/or microbial survival in the silk environment, whole-genome sequencing and candidate gene mining were undertaken for representatives of the most prevalent species from healthy silks along with additional bacteria that were selected to expand the taxonomic diversity or based on the literature. In total, 25 bacteria were mined, of which a subset were functionally tested in vitro, including the most prevalent species.

### 3.2. Water Limitation-Associated Traits

**Figure 2:**
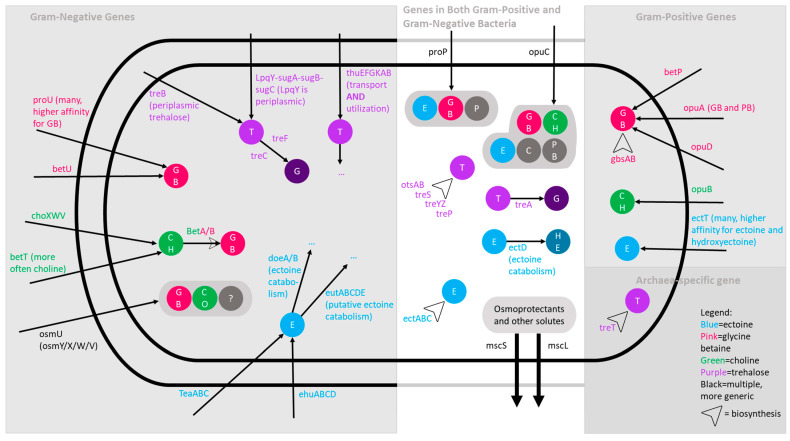
Genes involved in osmoprotectant transport and utilization. Genes that appeared to be relevant to this research were selected from the literature. Abbreviations: T: trehalose; GB: glycine betaine; CH: choline; E: ectoine; G: glucose; P: proline; HE: hydroxyectoine; PB: proline betaine; C: carnitine; CO: choline-O-sulfate; ?: possibly other osmoprotectants. Proline-specific transporters were not included, because proline plays many roles in cells and is non-specific to osmoprotection. The 3 dots (…) indicates that the metabolite is broken down into further metabolites. This is not a complete account of all osmoprotectants or osmoprotectant transport/utilization genes, as this is a dynamic area of research, and many genes have been discovered recently or are predicted to exist but not yet identified.

**Figure 3:**
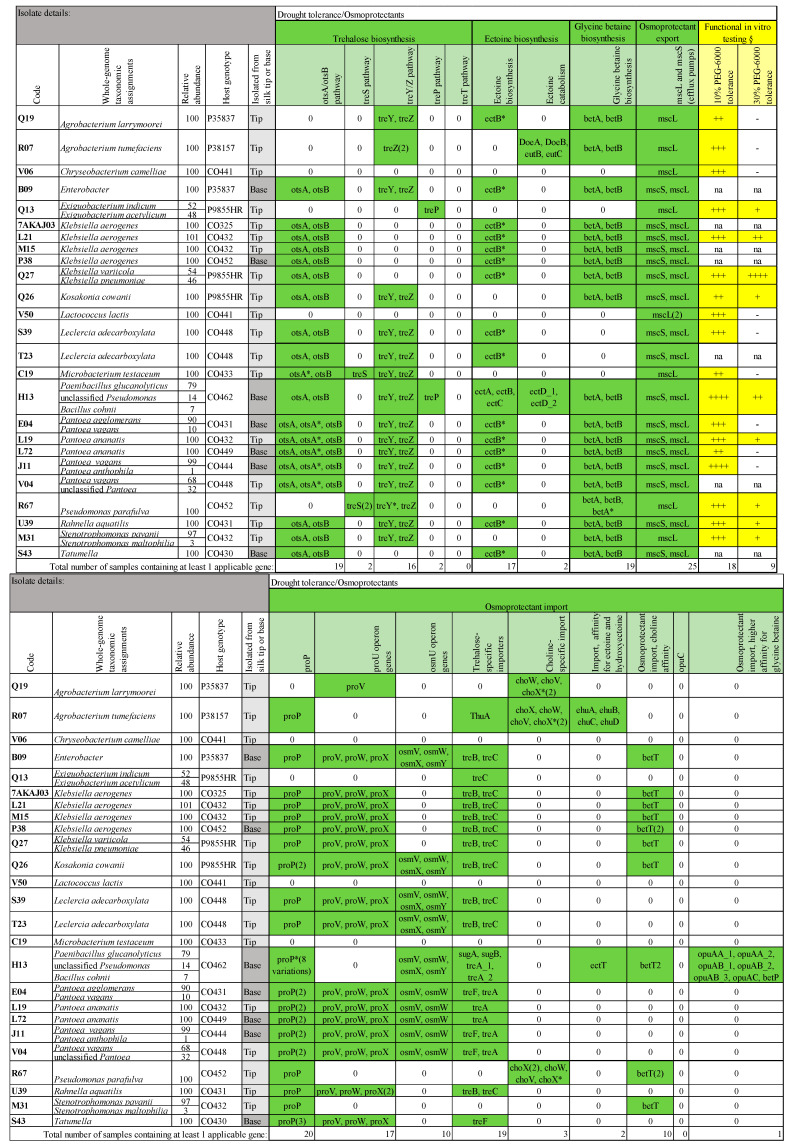
Summary of whole-genome mining for genes involved in osmoprotectant transport and utilization and results of functional in vitro tests for PEG-6000 tolerance (mimicking desiccation) for the most prevalent and select isolates from the cultured transmitting silk microbiome of maize. Silk samples spanned diverse host inbred/hybrid lines and heterotic groups of maize. Yellow cells indicate positive in vitro results. Green cells indicate the presence of relevant genes. The asterisk (*) indicates a likely match to the indicated gene. § indicates the resistance to PEG-6000 in terms of growth (calculated as the ratio of bacterial growth with PEG-6000/without PEG-6000 based on the average of 3 OD_600_ readings) and is grouped into the following ranges: - (not resistant, 0.00–0.08), + (mild resistance, 0.08–0.12), ++ (moderate resistance, 0.12–0.31), +++ (moderate-high resistance, 0.31–0.50), and ++++ (high resistance, >0.50). The “na” indicates that the isolate was not tested.

Since silk growth is limited by water availability [34], and since exposed silks can dry out, we hypothesized that silk microbiota may contribute to silk health and/or their own survival by synthesizing and exporting/importing osmoprotectants including trehalose, glycine betaine, ectoine, carnitine, proline betaine, and proline [35,36] (Figure 2 and Figure 3). Below, we describe the genes discovered for these osmoprotectants and the results of functional testing for tolerance to PEG-6000 in vitro (Figure 3). For the gene mining, proline-specific genes were excluded, because as an amino acid, proline is involved with many cellular processes [37]. Carnitine biosynthesis has not been observed in bacteria [38]. In general, osmoprotectant exporters are theorized in bacteria but are mostly not identified, with the exception of the *msc* system below:

*Osmoprotectant export genes:* The genes *mscL* and *mscS* encode mechanosensitive channels that play important roles in the bacterial export of solutes and other osmoprotectants, particularly under osmotic shock [39]. The *mscL* gene is nearly ubiquitous in bacteria [39] and was found in all 25 genomes analyzed, while *mscS* was detected in 17 bacteria.

*Trehalose biosynthesis and import genes:* Candidate genes involved in four common trehalose biosynthesis pathways (*otsAB*, *treS*, *treYZ*, and *treP*) [40,41] were identified (Figure 3). Of these, *otsA*/*otsB* were detected in 19 of the 25 bacteria, *treY*/*treZ* in 16 bacteria, *treS* in 2 bacteria, and *treP* also in 2 bacteria. No genes were found for a fifth pathway (*treT*) previously found in some primitive bacteria [41,42]. The gene *treB,* involved in the movement of trehalose from the bacterial periplasm to the cytoplasm [43,44], was identified (10 bacteria), along with genes (*treA*, *treC*, and *treF)* from multiple pathways involved in converting trehalose into glucose [*treA* (6 bacteria), *treC* (11 bacteria), and *treF* (4 bacteria)] [43,45]. With respect to the bacterial uptake of trehalose by the LpqY-sugA-sugB-sugC recycling transporter, *sugA* and *sugB* were only detected in one isolate (H13) [46]. Regarding the trehalose transport and utilization operon *thuEFGKAB* [47], only one bacterium encoded the trehalose utilization gene *thuA*.

*Ectoine biosynthesis and import genes:* With respect to ectoine, within the biosynthesis *ectABC* operon [48], *ectA*, *ectB*, and *ectC* were found in one bacterium (isolate H13), while potential matches for *ectB* were found in sixteen other isolates (Figure 3). Isolate H13 also contained *ectT*, an importer for multiple osmoprotectants, with a higher affinity for ectoine and hydroxyectoine [49]. No genes were detected for the osmoregulated transporter TeaABC, which imports ectoine [50,51,52]. There are additional ectoine-specific import genes (*ehuABCD*) that share an operon with catabolism genes (*eutABCDE*) [53]; the genes *doeA* and *doeB* are also involved in ectoine catabolism [54,55]. All of the latter ectoine import genes (*ehuA*, *ehuB*, *ehuC*, and *ehuD*) and several of the catabolism genes (*eutB*, *eutC*, *doeA*, and *doeB*) were detected in one isolate, R07. The *ectD* gene is also involved in ectoine catabolism (produces hydroxyectoine) [55,56], and one isolate (H13) contained two *ectD* genes.

*Glycine betaine biosynthesis and import genes:* The genes *betA* and *betB* are involved in converting choline to glycine betaine [36] and were found in 19 genomes. The biosynthesis enzymes *gbsB* (a type III alcohol dehydrogenase) and *gbsA* (a glycine betaine aldehyde dehydrogenase) (operon *gbsAB*) also transform choline into betaine [36], but these genes were not found. Glycine betaine-specific importers include *betU*, *betP*, *opuD*, the *opuA* transport system operon (genes *opuAA, opuAB,* and *opuAC*), and the *osmU* operon (genes *osmY*, *osmX*, *osmW*, and *osmV*, responsible for importing glycine betaine, choline-O-sulfate, and possibly others) [35,36,57,58,59,60,61]. Ten of the genomes contained *osmU* genes, five of which contained all four genes (*osmY*, *osmX*, *osmW*, and *osmV*), while one isolate (H13) notably had genes for two routes of import for glycine betaine (*betP* and the *opuA* transport system).

*Choline biosynthesis and import genes:* Choline is a precursor for glycine betaine, but choline itself is not an osmoprotectant [62,63]. The genes *betT*, *opuB*, and *choXWV* import choline into bacteria [36,64,65], and of these, *betT* was found in 10 genomes, *choW, choV,* and *choX* were found in 3 genomes, while *opuB* was not identified. Choline biosynthesis does not appear to be clearly identified in bacteria.

*Promiscuous osmoprotectant import genes:* Contrasting the more substrate-specific transporters, some transporters import a variety of osmoprotectants. The proP transporter imports most osmoprotectants, including ectoine, glycine betaine, and proline [36,66,67,68,69]. The majority of silk-associated bacteria sequenced (20/25 genomes) included at least one copy of the *proP* gene or a likely *proP* variant. The proU transporter has a higher affinity for glycine betaine but also imports most osmoprotectants, including choline, carnitine, proline betaine, and ectoine and includes the genes *proV*, *proW,* and *proX* [36,59]. Sixteen of the genomes contained all three *proU* genes, and an additional genome contained only *proV*. The opuC transporter imports the osmoprotectants glycine betaine, choline, carnitine, proline betaine, and ectoine [36,65] and was not detected in any of the genomes.

*In vitro testing of silk-associated bacteria under water limitation:* All 18 isolates tested grew moderately at 10% PEG-6000 [14–64% of growth (OD_600_) compared to growth without PEG]. Of the 12 most prevalent isolates, 5 also grew at 30% PEG-6000: M31 (*Stenotrophomonas pavanii*), Q13 (*Exiguobacterium indicum-acetylicum*), L19 (*Pantoea ananatis*), L21 (*Klebsiella aerogenes*), and Q26 (*Kosakonia cowanii*) (Figure 3). Of the remaining six isolates tested, four grew at 30% PEG: R67 (*Pseudomonas parafulva*), U39 (*Rahnella aquatilis*), H13 (*Paenibacillus glucanolyticus*), and Q27 (*Klebsiella variicola-pneumoniae*) which was the top performer in the collection, growing better at 30% PEG than without PEG.

Of the 18 isolates that were tested for PEG tolerance, all 6 that contained a *betT* gene (associated with choline import) were resistant to 30% PEG-6000. Two isolates contained *treP* (a trehalose biosynthesis pathway), both of which were resistant to 30% PEG-6000. Of the nine isolates that were resistant to 30% PEG-6000, eight contained *proP* (osmoprotectant import), eight contained *betA* and *betB* (glycine betaine biosynthesis), seven contained *otsA/B* (a trehalose biosynthesis pathway), seven contained at least one gene for trehalose import, six contained *treY/Z* genes (a trehalose biosynthesis pathway), five contained proU operon genes, five contained *ectB* (ectoine biosynthesis), and three contained osmU operon genes (glycine betaine and choline import). Two isolates were noteworthy: H13 and R67. Isolate H13, which was resistant to 30% PEG-6000, contains the *treP* trehalose biosynthetic gene and also uniquely contains genes associated with glycine betaine import, ectoine and hydroxyectoine import, unique trehalose-specific importer genes, eight variations of *proP*, *ectD-1*, and *ectD-2* genes for ectoine catabolism, and all three ectoine biosynthesis genes (*ectA, ectB,* and *ectC*). Isolate R67 was moderate to highly resistant to 10% PEG-6000 and mildly resistant to 30% PEG-6000; it contains choline-specific importer genes, and uniquely contains two copies of *treS* (a trehalose biosynthesis pathway).

### 3.3. Indole Compound-Associated Traits (Auxin Biosynthesis)

**Figure 4:**
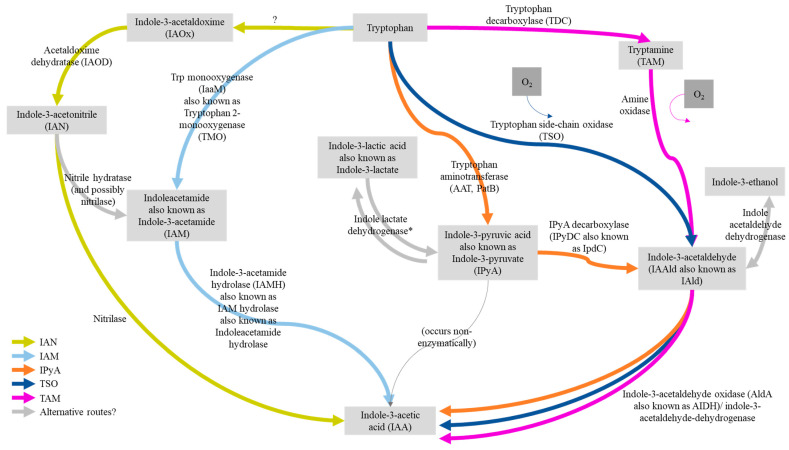
Summary of bacterial indole-3-acetic acid biosynthesis pathways. Pathways and genes that appeared to be relevant to this research were selected from the literature. The asterisk (*) indicates that the gene was not reported in GenBank. This is a dynamic area of research, and some genes have been predicted to exist but not yet identified. The question mark (?) indicates that the step is unknown. Based on works by D. R. Duca and Glick [72], Keswani et al. [73], Kunkel and Harper [74], Morffy and Strader [75], Shao et al., [76,77], Spaepen, Vanderleyden, et al. [78], Spaepen, Versées, et al. [79], and Spaepen and Vanderleyden [80].

**Figure 5:**
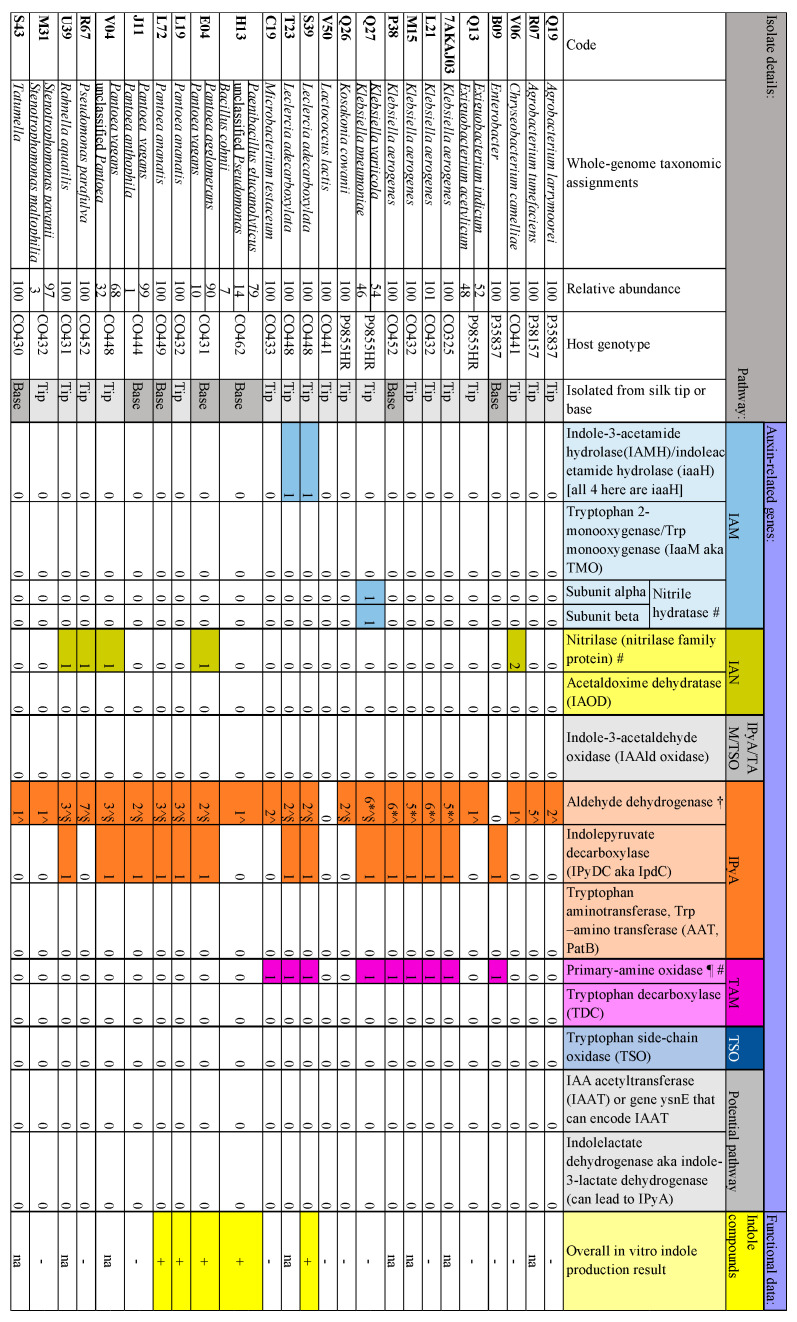
Summary of whole-genome mining results for genes involved in indole-3-acetic acid biosynthesis and results of functional in vitro testing for production of indole-containing compounds (3 replicates) for the most prevalent and select isolates from the cultured transmitting silk microbiome of maize. Silk samples spanned diverse host inbred/hybrid lines and heterotic groups of maize. † There were no exact matches for indole-3-acetaldehyde dehydrogenase, IAAld dehydrogenase, or IAld dehydrogenase (AIDH, also called AldA), but various genes are labeled more generically as aldehyde dehydrogenase. ¶ Amine oxidase is a putative step in the pathway, which is not well defined, and the genes found in this study are primary-amine oxidases. Symbol definitions: # gene family, not specific to this pathway; * acetaldehyde dehydrogenase (acetylating); ^ aldehyde dehydrogenase; § aldA (aldehyde dehydrogenase). The addition symbol (+) indicates that the isolate convincingly produced indole compounds in 3/3 replicates. The subtraction symbol (-) indicates that the isolate did not meet these requirements. The “na” indicates that the isolate was not tested.

The plant hormone auxin is involved in stimulating plant growth, and plant auxin signaling and regulation transcripts are abundant in maize silks [70,71]. Furthermore, maize pollen is abundant in the auxin, indole-3-acetic acid (IAA), and delivers IAA to silks upon pollination, where it stimulates egg cell maturation [70]. Bacteria encode five auxin biosynthetic pathways (Figure 4 and Figure 5) [72,73,74,75,76,77,78,79,80]. Within the 25 bacterial genomes, genes from 4 IAA biosynthetic pathways were identified (Figure 5).

*IAM pathway:* In the indole-3-acetamide (IAM) pathway [72,81,82], the gene *iaaH* (indoleacetamide hydrolase) was found in two bacteria, and the genes *nthA* and *nthB* (nitrile hydratase subunits alpha and beta) were found in one bacterium. Nitrile hydratase can connect the IAM and IAN pathways [81].

*IAN-specific pathway:* In the IAN (indole-3-acetonitrile) pathway [81], the genes for a nitrilase family protein were found in five bacteria, specifically belonging to Class 13 nitrilases [83]. Pseudo genes listed as “nitrilase family protein” were not counted (B09 and L17).

*IPyA, TAM, and TSO pathways: pathway-specific upstream enzymes:* In the indole-3-pyruvic acid (IPyA) pathway, the gene for indolepyruvate decarboxylase (sometimes annotated as *ipdC*) was found in 14 bacteria [84]. Thirteen of the genomes contained both a version of acetaldehyde dehydrogenase and indole-3-pyruvic acid decarboxylase, which are both involved in the IPyA pathway. An alternative route to IPyA involves indole lactate dehydrogenase converting indole-3 lactate into IPyA and vice versa [72], although this enzyme was not detected in any of the genomes here. In the tryptamine pathway (TAM) in Gram-negative bacteria, a putative amine oxidase is required [72,76,85,86]; candidate primary-amine oxidases were found in nine bacteria. In the tryptophan side-chain oxidase (TSO) pathway [72,87], no genes encoding a TSO enzyme were detected in the genomes.

*IPyA, TAM, and TSO pathways: shared downstream enzymes:* There is a common enzyme in the IPyA, TAM, and TSO pathways that transforms indole-3-acetaldehyde into IAA: some studies report this as indole-3-acetaldehyde dehydrogenase (AIDH, also called *AldA*), while others report it as indole-3-acetaldehyde oxidase [72,82,85,87,88]. A report from Duca and Glick suggests that AIDH converts indole-3-ethanol to indole-3-acetaldehyde [72]. No indole-3-acetaldehyde dehydrogenase annotated genes were found, but the bacteria encoded a generic aldehyde dehydrogenase: 11 were annotated as *aldA* (not to be confused with capitalized AldA), and 23 were annotated only as ‘aldehyde dehydrogenase’; another 5 were annotated as ‘acetaldehyde dehydrogenase (acetylating)’. It should be noted that aldehyde dehydrogenases are not specific to the IAA pathway.

*Alternative route:* IAA acetyltransferase (IAAT), which may be encoded by the gene *ysnE*, may be involved in another Trp-dependent IAA synthesis pathway [73] but was not detected in the genomes.

*In vitro testing of silk-associated bacteria for indole compound production:* Of the 17 isolates tested, 5 convincingly produced indole compounds (3/3 replicates): E04 (*Pantoea agglomerans*), L19 (*Pantoea ananatis*), L72 (*Pantoea ananatis*), S39 (*Leclercia adecarboxylata*), and H13 (*Paenibacillus glucanolyticus*). Of these, all five contained at least one copy of the gene encoding indole-3-acetaldehyde dehydrogenase, four encoded indolepyruvate decarboxylase, and one encoded a nitrilase family protein. S39 (positive for indole production) was the only sample tested that encoded indole-3-acetamide hydrolase. S39 also contained one amine oxidase gene, making it a total of three potential IAA-production pathways implicated in its genome.

### 3.4. Nitrogen Limitation Traits

**Figure 6:**
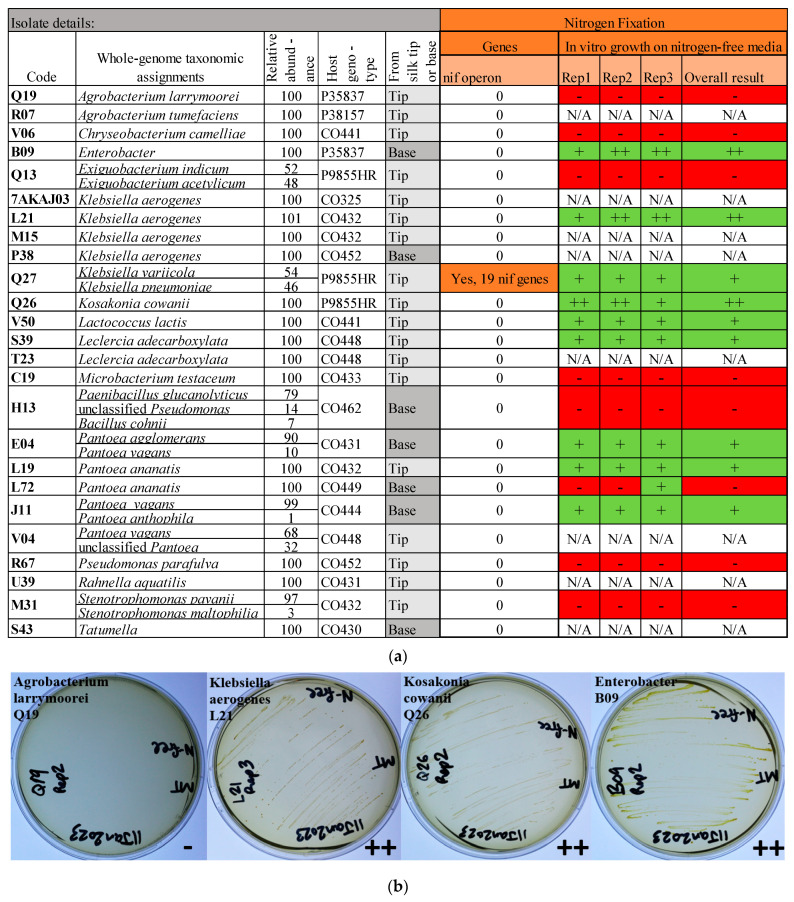
Summary of (**a**) whole-genome mining for genes involved in nitrogen fixation and (**b**) results of functional in vitro results for growth on nitrogen-free media for the most prevalent and select isolates from the cultured transmitting silk microbiome of maize. Silk samples spanned diverse host inbred/hybrid lines and heterotic groups of maize. Nitrogen-free growth testing occurred in an anaerobic chamber, and the results indicate continued growth after restreaking from an initial nitrogen-free plate, all within an anaerobic chamber. Candidates were considered positive if they grew on both the first and second set of restreaked plates, in 3/3 replicates. The symbol “-” in red cells indicates no growth, in green cells “+” indicates growth, and “++” indicates strong growth. The “na” indicates that the isolate was not tested.

Since silk growth is limited by nitrogen [89], we screened the silk-associated bacterial genomes for nitrogen fixation (*nif*) genes (Figure 6). Only 1 of the 25 isolates, P1-19CT-C (Q27) (*Klebsiella variicola*/*pneumoniae*), contained a suite of *nif* genes (*nifABDEHJKLMNQSTUVWXYZ*). The gene *nifJ*, which is not specific to nitrogen fixation, was found in 11 of the genomes.

*In vitro testing of silk-associated bacteria under nitrogen limitation:* Of the 17 isolates tested, 9 grew on the second restreak onto nitrogen-free media (pre-reduced for oxygen, in anaerobic chamber): J11 (*Pantoea vagans*), E04 (*Pantoea agglomerans*), L19 (*Pantoea ananatis*), L21 (*Klebsiella aerogenes*), Q26 (*Kosakonia cowanii*), V50 (*Lactococcus lactis*), Q27 (*Klebsiella variicola*/*pneumoniae*), S39 (*Leclercia adecarboxylata*), and B09 (*Enterobacter*). Of these, isolate Q27 was confirmed to contain a suite of *nif* genes.

### 3.5. Pollen/Fertilization Signaling Traits

Bacteria in the silks/pollen tubes may play a role in signaling, with molecules including GABA, ethylene, and nitric oxide.

**Figure 7:**
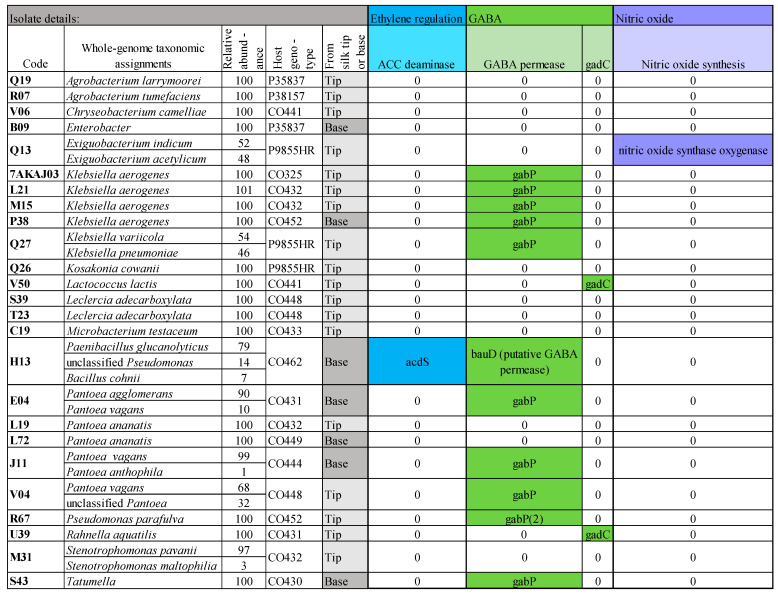
Summary of whole-genome mining results for genes involved in pollen/fertilization signaling compounds for the most prevalent and select isolates from the cultured transmitting silk microbiome of maize. Genes include those related to ethylene, gamma-aminobutyric acid (GABA), and nitric oxide. Silk samples spanned diverse host inbred/hybrid lines and heterotic groups of maize.

*GABA genes*: Gamma-aminobutyric acid (GABA, γ-aminobutyrate) is a signaling molecule involved in stimulating and guiding pollen tube elongation towards the ovule based on a concentration-dependent gradient [90,91]. Genes involved in GABA production (*gadR*, *gadC*, *gadB*, *gts*, and *gadA*) have been identified in lactic acid bacteria [92,93]. The gene *gadC* (glutamate:gamma-aminobutyrate antiporter) permits the export of GABA via the uptake of glutamate [94]. Here, *gadC* was found in two silk-associated bacteria, *Lactococcus lactis* (V50) and *Rahnella aquatilis* (U39) (Figure 7). Additionally, 11 bacteria contained GABA permease (*gabP*), which imports GABA [95] and, hence, could theoretically modulate the host GABA concentration. The other *gad/gts* genes were not found here.

*ACC deaminase genes*: In maize silks, pollination induces the synthesis of the plant hormone ethylene, which then promotes egg cell maturation, while blocking ethylene has the opposite effect [70]. Additionally, ethylene signals senescence [96,97]. Bacteria can secrete the enzyme ACC deaminase, which cleaves the precursor of ethylene, 1-aminocyclopropane-1-carboxylic acid (ACC), thus reducing the plant ethylene concentrations [98,99] and possibly extending or regulating the pollination window. The structural gene encoding ACC deaminase (*acdS*) [98] was detected in the genome of one transmitting silk bacteria (H13) (Figure 7). Other ACC-related genes such as ACC deaminase regulatory gene (*acdR*) and ACC synthase [100,101] were not detected in the bacterial genomes here.

*Nitric oxide genes*: The signaling molecule nitric oxide (NO) has been shown to alter the rate and direction of pollen tube growth [102,103]. Nitric oxide synthase oxygenase was detected in the genome of one transmitting silk-associated bacteria (Q13) (Figure 7). Other NO-associated genes, such as *nirS*, heme-based monooxygenase, pterin cofactor H_4_B, and THF synthesis were not found [104,105,106].

### 3.6. Aluminum Tolerance Traits

**Figure 8:**
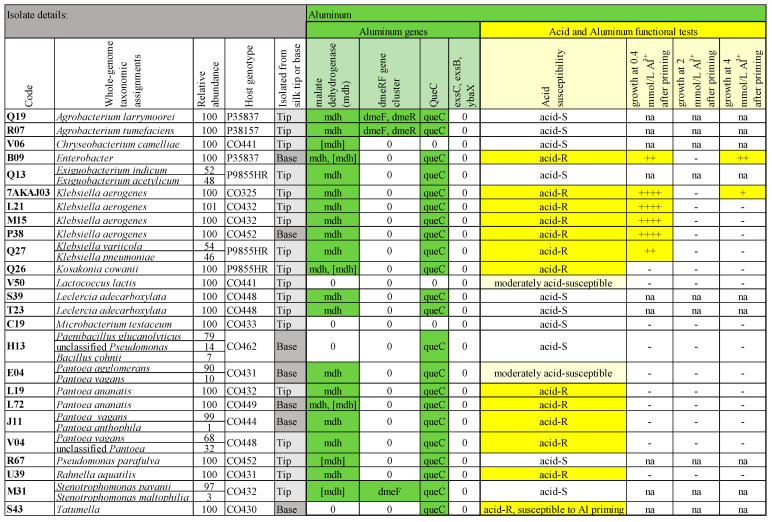
Summary of whole-genome mining results for aluminum-related genes and results of functional in vitro testing for aluminum and acid tolerance for the most prevalent and select isolates from the cultured transmitting silk microbiome of maize. Silk samples spanned diverse host inbred/hybrid lines and heterotic groups of maize. The functional data are the average of 3 replicates for each aluminum concentration. Resistance to aluminum was measured in terms of growth (calculated as the ratio of bacterial growth with aluminum/without aluminum based on the average of 3 OD_600_ readings) and grouped into the following ranges: - (not resistant, 0.00–0.08), + (mild resistance, 0.08–0.12), ++ (moderate resistance, 0.12–0.31), +++ (moderate-high resistance, 0.31–0.50), and ++++ (high resistance, >0.50). The “na” indicates that the isolate was not included in the specific test.

Maize pollen has high concentrations of aluminum [19], and since bacteria have been shown to reside on the pollen surface [107], we hypothesized that any bacteria in transmitting silks that originated from pollen might possess genes for aluminum tolerance. Despite various claims in the literature, we only included Al-tolerance genes that were backed by functional studies (Figure 8).

Malate dehydrogenase synthesizes malate, a precursor for citrate, and these organic acids promote aluminum tolerance; the underlying gene *mdh* is upregulated in aluminum-tolerant plant-associated bacteria when treated with aluminum [108,109,110]. Twenty-one of the genomes contained malate dehydrogenase, eighteen of which were annotated specifically as *mdh* (Figure 8).

The heavy metal-inducible *dmeRF* gene cluster is suggested to contribute to endophyte tolerance of metals, perhaps including aluminum [111,112]. The divalent metal efflux gene (*dmeF*) and the open reading frame/transcriptional repressor (*dmeR*) were found together in two bacteria, and *dmeF* was found alone in another bacterium (Figure 8).

Aluminum resistance proteins G2alt and ALU1-P [33,113] were not found in the genomes (Figure 8). However, ALU1-P is a member of the QueC family [33,114]. This family encodes 7-cyano-7-deazaguanine synthase, the first step in the queuosine biosynthetic pathway, and was found in 22 of the genomes, but it is not clear whether the immediate product promotes aluminum tolerance or is just a precursor and, hence, is non-specific.

*In vitro testing of silk-associated bacteria under acid and aluminum stresses:* The functional assay (adapted from Huang et al. [32] and Lim et al. [33]) for bacterial aluminum tolerance in GM broth required a pH of 3.5 to solubilize aluminum and, hence, inadvertently tested for acid tolerance in the 0% aluminum control. This was relevant to pollen tube-associated bacteria, since pollen tube tips secrete protons to facilitate growth and acidify the media in vitro [20,21,22,115]. Of the 25 isolates tested, 10–12 were resistant to pH 3.5 GM broth (Figure 8). The assay required sequential priming to initial low concentrations of aluminum (50 µmol/L, then 0.1 mmol/L); at this stage, 10 of the isolates failed to grow. Of the twelve isolates that showed sustained resistance to acid and Al priming, six grew at 0.4 mmol/L Al^3+^ [16–96% of growth (OD_600_) compared to growth without Al^3+^] and two isolates grew at 4 mmol/L Al^3+^, one of which, B09 (*Enterobacter*), showed moderate growth (14%). All six bacteria that showed resistance to acid and aluminum contained *mdh* and *queC* (Figure 8).

## 4. Discussion

### 4.1. Overview

The fertilization stage at which male gametes are transmitted to the ovule in the style/silks (transmitting stage) is a critical stage in reproduction. Here, genome mining and functional testing of bacteria cultured from the transmitting silk microbiome (TSM) revealed evidence of signaling traits including auxin, GABA, NO, and ACC deaminase, all implicated in successful host fertilization. Further evidence suggested that members of the TSM are tolerant to silk- and pollen-relevant stressors, including water and nitrogen limitation, and acid and aluminum toxicity.

### 4.2. TSM Traits in the Context of Vertical Transmission

The male and/or female gamete may transmit endophytes to the offspring [15,116]. It has been suggested that endophytes may be inherited via the pollen tube [13,14,116]. A study by Liu et al. [10] showed evidence of hybrid maize offspring receiving members of their microbiome from both the male and female parents. Wu et al. [12] recently demonstrated that maize pollen carries a *Bacillus mojavensis* strain that can be transmitted to progeny seed. The pollinated silks in the current study potentially contain pollen tubes and migrating male gametes.

**Figure 9:**
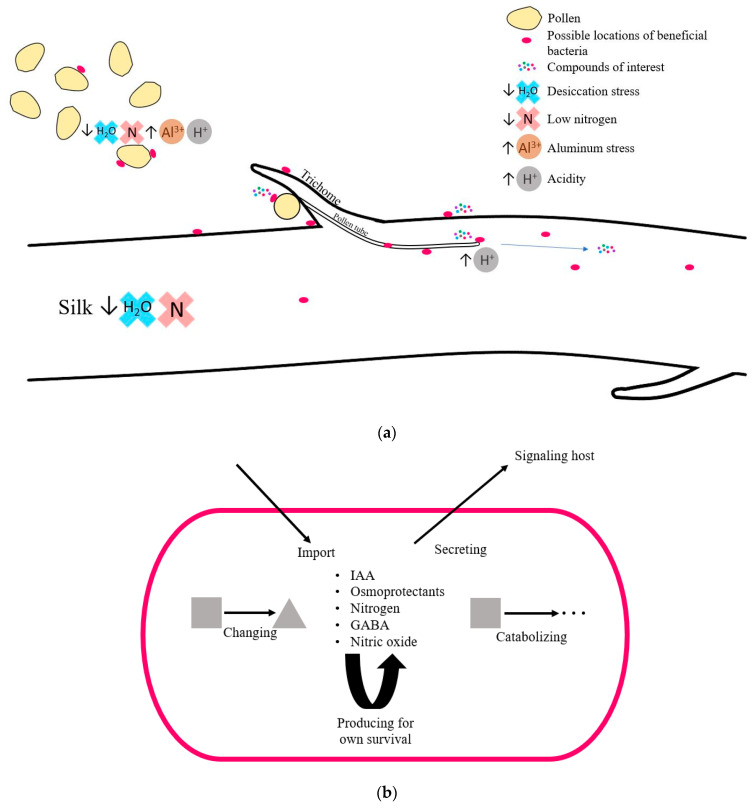
Proposed summary model of (**a**) possible locations of bacteria and stressors that bacteria encounter in the context of style tissue, pollen, and pollen tubes of fertilization-stage maize silks and (**b**) possible interactions of predicted stress tolerance and signaling metabolites associated with bacteria in fertilization-stage silks. ACC refers to 1-aminocyclopropane-1-carboxylic acid, the precursor of ethylene. GABA refers to gamma-aminobutyric acid.

Therefore, fertilization-stage silks presumably contain maternally derived silk-associated microbes along with paternally derived (pollen) microbes associated with the pollen tubes and male gametes. Vertically transmitted pollen-associated bacteria would either need to colonize the male gametes or the leading edge of the pollen tube (Figure 9). By contrast, vertically transmitted maternal host bacteria would either colonize the ovule directly or transfer onto the pollen tube as it grows through the silk channel. This poses two challenges for such vertically transmitted microbes: surviving the unique stresses of the pollen and/or silk environment and promoting host reproductive success to ensure their own survival or the survival of their genetically related siblings, like worker bees in a colony functioning as a superorganism to help their queen proliferate [117].

### 4.3. Tolerance of the TSM to Silk/Pollen Abiotic Stresses

Both the silks and the pollen tectum (outer surface) can be a place of water and nitrogen limitation. Pollen, in particular, undergoes dehydration and shriveling (infolding of the sporoderm aperature) [118] before rehydrating on the surface of silks [2]. Pollen is also relatively high in aluminum [19], which can be toxic to bacteria [119]. As well, the leading edge of the pollen tube is believed to have a high concentration of hydrogen ions, making it a locally acidic environment [21,22,120]. In this context, it is noteworthy that the TSM is abundant in Gammaproteobacteria, which are known to survive in diverse, sometimes extreme environments [121,122]. Furthermore, the TSM is rich in Pseudomonadota, which includes many nitrogen fixers [123]. Here, culturing permitted detailed genome mining and phenotyping. We found support for the hypothesis that members of the TSM are tolerant of the stresses common on silks/pollen:

#### 4.3.1. Desiccation Tolerance

Functional desiccation tolerance tests revealed that all 18 TSM isolates tested grew in 10% PEG-6000 and 9 grew in 30% PEG-6000, providing functional evidence of desiccation tolerance in the TSM. Gene mining also supported this hypothesis, with evidence for osmoprotectant biosynthesis in 23/25 isolates, osmoprotectant import in 22/25 isolates, and possible osmoprotectant efflux (*mscL* and *mscS*) in all 25 isolates. The export of osmoprotectants leaves open the intriguing possibility that TSM bacteria may be providing osmoprotectants to benefit the host plant, including reproductive tissues. Bacterial osmoprotectant export (secretion and efflux) is an area of active research, and there are major export routes that have not yet been identified [124,125].

There were 15 isolates that contained genes implicated in different biosynthesis pathways for the osmoprotectant trehalose, indicating their ability to use different substrates to produce trehalose. This potential redundancy may imply the bacteria’s ability to produce trehalose under different conditions. Likewise, one isolate (H13) contained biosynthesis genes (*ectA, ectB,* and *ectC*) for the osmoprotectant ectoine and encoded an import protein with an affinity for ectoine (EctT). Ectoine can be synthesized or imported by bacteria, and some bacteria, like *Halomonas elongata,* do both [125].

Some bacteria can use the osmoprotectants glycine betaine and choline additionally as sources of nitrogen and carbon [62]. Bacteria in the Rhizobiaceae family metabolize glycine betaine for energy, and only specific strains utilize glycine betaine as an osmoprotectant [62]. Bacteria from the TSM may be feeding off of glycine betaine and choline produced by the host or by other bacteria. Maize produces trehalose and glycine betaine at the silking stage [126], so it is possible that these are present in the silks. Choline is biosynthesized and exuded by plants and utilized by symbiotic microbes [127]. Maize also can accumulate choline [128], so the silk bacteria with choline import and utilization genes may be harvesting host-produced choline.

After successful pollination, an abscission zone forms in the silk, essentially cutting off the tissue from its connection to the vascular system, causing the silks to dehydrate and senesce [23,24]. This brings into question whether TSM bacteria require these mechanisms to survive on senescing silks.

#### 4.3.2. Nitrogen Limitation

Functional tests showed nine TSM isolates had convincing nitrogen-free anaerobic growth. Curiously, only 1 of the 25 isolates contained a suite of *nif* genes. However, this may not be surprising, because *nif* operons are often found on plasmids [129], which are sometimes excluded from whole-genome sequencing. The sole isolate containing *nif* genes was *Klebsiella variicola*/*pneumoniae*; *Klebsiella* species, especially *K. variicola* in maize, are well-known nitrogen fixers and believed to be transmitted by maize seeds [130,131,132,133]. Our data suggest that nitrogen-fixing *Klebsiella* are colonizing pollinated silks. Molybdenum, a cofactor for nitrogenase, is present in maize pollen [19,134]. This opens doors for further studies investigating whether TSM bacteria contribute to nitrogen fixation in the silks, pollen tubes, or pollen, or are simply transmitted to seeds to supply nitrogen to other progeny tissues such as the maize xylem sap [135].

#### 4.3.3. Aluminum Tolerance

Bacteria have been previously shown to live on pollen [107], which, in maize, is unusually high in aluminum [19]. Many plant growth-promoting microbes have been identified as inducing the overexpression of aluminum tolerance genes by the plant host (some perhaps working with IAA, etc.) [136]. Alternative to the pollen–aluminum concept, aluminum tolerance may help the bacteria (and host) survive high-aluminum soil environments [137]. Aluminum tolerance can also be associated with cell wall thickness [138]. Several TSM bacteria in the functional tests appeared to be aluminum tolerant: six isolates grew in 0.4 mmol/L Al^3+^, and two grew at 4 mmol/L Al^3+^. Gene mining revealed all of the positive candidates contained the aluminum tolerance genes *mdh* and *queC*, although these genes may be involved in other cellular processes [130,139]. The three isolates containing *dmeR/F*, a gene cluster suggested to help metal tolerance by endophytes [111,112], were not resistant to the acidity in the assays; thus, their aluminum tolerance could not be assessed.

#### 4.3.4. Acid Tolerance

The pollen tube tip secretes protons in the process of extending through the silks, so microbes attempting to enter seed with the male gamete may need to tolerate this slightly acidic environment [20,21,22,115]. In this context, it was interesting that the acidic aluminum tolerance assay revealed that 13/25 of the tested TSM bacteria were tolerant to a pH of 3.5.

### 4.4. Evidence for Possible Selection on the TSM for Signaling Compounds That Promote Host Reproduction

We hypothesized that bacteria inhabiting fertilization-stage silks may promote host reproductive success to ensure their own survival. In fruit flies and mammals, bacteria promote successful mating [16,17]; however, it is unknown whether bacteria play a beneficial role in plant reproduction. Here, members of the TSM were found to encode signaling metabolites known to be involved in plant reproduction:

#### 4.4.1. Auxin Biosynthesis

Maize pollen is abundant in the auxin, indoleacetic acid (IAA), and delivers IAA to silks upon pollination, where it stimulates egg cell maturation [70]. Auxin RNAs are also overexpressed in silks [71], presumably to promote growth. Five of the seventeen tested TSM isolates were positive for indole production. Combining the 25 TSM genomes analyzed, genes implicated in four IAA biosynthetic pathways were identified; at least 1 gene was found in 24/25 isolates. Thirteen of the genomes contained both acetaldehyde dehydrogenase and indole-3-pyruvic acid decarboxylase, which are both involved in the IPyA pathway for biosynthesis of IAA [78]. The in vitro assay detected strong production of indole compounds from 5/17 tested isolates, although less intense IAA producers were likely missed because the color-based test was not particularly sensitive. It is also worth mentioning that although individual genes involved in IAA production were detected, these genes are involved in complex pathways that rely on multiple genes (steps) to reach the final product. It is possible that the bacteria did contain a complete set of genes required for IAA production (some could have been missed in genome sequencing/processing), but the bacteria did not produce detectable amounts or did not produce IAA under the non-native assay conditions. It is also a possibility that the bacteria did not contain all of the genes necessary to complete a pathway for IAA production independently. Endophytes and plant hosts are known to share secondary metabolites, for example, the taxol pathway in *Taxus* (yew) trees [140,141]. It may be that TSM bacteria and host maize exchange IAA and/or share IAA biosynthetic pathway intermediates. Indeed, it is hypothesized that the IPyA pathway functions in both bacteria and plants [78], with the enzyme indolepyruvate decarboxylase being a rate-limiting step [142], leaving open the possibility that the 14 TSM isolates identified here to encode *ipdC* may be collaborating to synthesize IAA, possibly in maize reproductive tissues. Alternatively, these microbial genes may be acting in host progeny tissues (e.g., roots) [82].

#### 4.4.2. GABA

Gamma-aminobutyric acid (GABA, γ-aminobutyrate) is involved in tube attraction to an ovule by forming a concentration-dependent gradient [90,91]. Both GABA importers and exporters may be involved in regulating the concentration gradient of GABA in transmitting silks. Here, the GABA permease (*gabP*) gene, which imports GABA, was found in 11 TSM genomes; previously, this gene was thought to be involved in repressing virulence gene expression [95], which could promote an endophytic lifestyle. GABA can also provide acid resistance to bacteria [143], which may help TSM endophytes adapt to the growing pollen tube (as mentioned above). In addition to GABA import, two TSM isolates, including *L. lactis*, had GABA export genes. This is consistent with a prior study showing that *Lactococcus* bacteria can produce high levels of GABA [143]. However, the TSM isolates may be encoding GABA import/export genes for other reasons. For example, GABA can be a food source for some bacteria [95]. Furthermore, in *Agrobacterium tumefaciens,* quorum sensing can be downregulated by GABA [144], though here, no GABA genes were found in the fully sequenced *A. tumefaciens* isolated from pollinated silks.

#### 4.4.3. ACC Deaminase

Ethylene increases in maize ears at pollination and promotes egg cell maturation [70]. Ethylene is also known as a signal for senescence [96,97], so reducing ethylene levels may extend the pollination window of silks, potentially contributing to host reproductive success. Here, we found evidence that at least one TSM isolate encodes ACC deaminase, which catabolizes the ethylene precursor [98]. Previously, the focus of this enzyme has been on the ability of root microbes to help host plants tolerate flooding stress [99], but perhaps ACC deaminase-encoding microbes play additional roles in plant reproduction.

#### 4.4.4. Nitric Oxide

Nitric oxide (NO) is a signaling molecule in animals and plants [145]. Here, the nitric oxide synthase oxygenase gene was detected in one TSM isolate. This was of interest, because nitric oxide regulates pollen tube growth and guidance [102,103]. In *Paulownia tomentosa*, UV-B treatment of pollen decreased germination and pollen tube growth and increased NO synthesis [146]. He et al. [146] suggest that UV-induced pollen inhibition is partly caused by NO synthesis. In general, bacterial nitric oxide synthases are involved in adapting to oxidative stress, repairing UV radiation damage, and acting as a protective agent, but have also been shown to promote the adhesion of a *Streptomyces* fungus to host plant roots [104,105,145]. In some *Streptomyces*, NO synthases are involved in nitrating plant toxins [147].

### 4.5. Some TSM Isolates Show Multiple Adaptations

Some important TSM isolates showed positive results for up to three functional tests. Two isolates [*Klebsiella aerogenes* (L21, OTU118) and *Klebsiella variicola/pneumoniae* (Q27, OTU144)] grew anaerobically without nitrogen and were resistant to 0.4 mmol/L Al^3+^ and to a high concentration of PEG-6000 (30%). Three other isolates [*Leclercia adecarboxylata* (S39, OTU167)], *P. agglomerans* (E04, OTU246), and *P. ananatis* (L19, OTU271, OTU276, OTU290, OTU292)] were PEG-tolerant (at 10%), produced indole compounds, and grew anaerobically without nitrogen. This latter result was of particular interest, because *P. agglomerans* and *P. ananatis* were amongst the most prevalent cultured TSM species [5,28].

### 4.6. Prior Studies Show Pantoea Can Benefit Plants and Be Vertically Transmitted

The genus *Pantoea* is prevalent and diverse in the TSM, including many strains of *P. agglomerans* and *P. ananatis* [5,28], which, as noted above, also displayed multiple adaptive traits relevant to host reproductive tissues in this study*. P. agglomerans* and *P. ananatis* have been found to cause growth promotion in maize [9,148]. *P. agglomerans* has been observed to produce IAA, solubilize phosphate [148], and induce salt tolerance [149] in maize. In green onion, a single strain of *P. agglomerans* can fix nitrogen in addition to promoting growth, solubilizing phosphate, and producing indole [150]. Maize-associated *Pantoea* have been found to fix nitrogen [151]. *Pantoea* found in *Zea* seeds have been shown to produce ACC deaminase [7]. In addition, *P. agglomerans* and *P. ananatis* have been used as biocontrols on the flowers of fruit trees [152,153,154,155] and to reduce the mycotoxin deoxynivalenol in wheat infected with *Fusarium graminearum* [156], respectively.

In general, *Pantoea* is ubiquitous across environments, a common plant colonizer, and includes many known epiphyte and endophyte species, as noted above [157]. *Pantoea*, at the genus level, have been observed in the germinated seeds [8], rhizosphere [148], roots, stems, and leaves [158] of maize. In support of vertical microbial transmission, genetically similar pathogenic *and* beneficial strains of *P. ananatis* have been isolated from healthy maize seeds [9]. *Pantoea,* intriguingly, are vertically transmitted in rice [11]. Xylanase may impact pollen grains getting into silk tissue, and a rice-endophytic *P. ananatis* has been shown to produce a xylanase-like compound [4,159].

### 4.7. Study Limitations and Future Experiments

A relatively small subset of the cultured healthy TSM was sequenced with WGS and used for gene mining and in vitro tests. Even before narrowing down the library of cultured isolates to the subset of bacteria used in this study, the library showed only a portion of the bacteria present in the TSM due to the limitations of culturing and sequencing (incompatibility with culturing conditions or primers). In general, the literature shows that culturing captures <10% of the bacteria present in a microbiome [160]. Nevertheless, we strove to include representatives of the most prevalent bacteria from healthy silks. Additional microbes from this library and other silk, pollen, and seed microbe collections should undergo gene mining and in vitro testing in the future.

In terms of whole-genome mining, there are many more genes which could be explored. Additionally, only a limited number of isolates underwent whole-genome sequencing due to cost. There are many more samples that may hold interesting genes. It is possible that certain genes were not identified due to low-depth sequencing. Furthermore, this is an emerging area of research, where some genes responsible for osmoprotectant transport and IAA synthesis are yet to be identified [73,124,125]. Additionally, genes (including nitrogen fixation genes) could be located on plasmids [129], which are sometimes excluded in this type of sequencing [161]. Likewise, silk/style bacteria could undergo further testing such as acetylene reduction assays. In addition to the potential impacts on maize reproduction presented in the current study, it is possible that the TSM may affect various agronomic traits in maize; we recommend that future breeding studies incorporate TSM analyses. Transcriptomics, proteomics, and metabolomics would also make interesting additions to future studies.

Aside from being essential for maize production, silks have been used as herbal or traditional medicine for hundreds of years, a feed supplement for stressed poultry, and contain various phytochemicals [162,163,164,165]. Some of these benefits perhaps come from the transmitting silk microbiome (TSM) [4]. A future area of study would be to phenotype and use multi-omics approaches on the cultured TSM microbes to investigate their ability to produce the phytochemicals previously attributed to silks.

This study focused on pollinated silks, but moving forward, unpollinated silks and pollen should be studied separately, as our group has begun [166,167], to resolve questions about the origin, inheritance, and roles of the TSM. In terms of future application, the bacteria identified here as providing potential benefits to host plants (e.g., nitrogen fixation) could be tested as future seed and/or field treatments. Furthermore, the identification of microbes that improve host reproductive success may allow breeders to use associated microbial markers for selection.

## 5. Conclusions

This was the first comprehensive functional study of a style microbiome at the fertilization stage (transmitting stage microbiome, TSM) in any plant species to the best of our knowledge. Microbes in the style tissue of other plant species should now also be investigated. Our results demonstrate value in culturing and testing potential roles of the microbiome, in addition to NGS. The findings suggest that members of the TSM, including from the core, encode adaptations to survive stress conditions of silk and pollen habitats, including water and nitrogen scarcity. Drought episodes are worsening due to climate change [168,169], while many resource-poor smallholder farmers cannot afford nitrogen fertilizer [170,171], both of which hinder reproductive success [18,25,26,172]; the extent to which these stresses will impact reproductive microbiomes and their consequences on host plants represent important areas of future study. This is especially the case because the results here demonstrate that members of the TSM have the potential to express signaling compounds known to impact reproduction. We propose that bacteria that are vertically transmitted secrete these compounds to promote host reproductive success to ensure their own survival. The concept that the microbiome may play a direct role in plant reproduction is a novel direction for future research.

## Data Availability

The whole-genome sequences corresponding to the strains in this study were deposited in the NCBI GenBank, and the accession numbers can be found in Figure 1b.

## Supplementary Materials

The following supporting information can be downloaded at: https://www.mdpi.com/article/10.3390/microorganisms12071473/s1, Figure S1: Whole-genome details; Supplementary Text S1: In vitro testing of microbial traits; Supplementary Text S2: Details for whole-genome sequencing, gene annotation, and mining.
