# Supplementary material for: The Microbiome of Fertilization-Stage Maize Silks (Style) Encodes Genes and Expresses Traits That Potentially Promote Survival in Pollen/Style Niches and Host Reproduction
Source: Microorganisms. 2024 Jul 19;12(7):1473. doi: 10.3390/microorganisms12071473 (PMC11278993; doi:10.3390/microorganisms12071473)
Supplement: Supplementary file 1 [file microorganisms-12-01473-s001.zip › microorganisms-3103773-supplementary.pdf]

Supplementary Materials

Code	ID	Whole-genome taxonomic assignment	Relative abundance	Accession number	Number of bases sequenced	Expected genome size	Calculated genome coverage	Illumina System	Company for sequencing
**Q19**	L1-17CT-A	*Agrobacterium larrymoorei*	100	SAMN37538683	4814147	5000000	0.963	Illumina NextSeq	MiGS
**R07**	P1-18CT-C	*Agrobacterium tumefaciens*	100	SAMN37538686	5712232	5600000	1.020	Illumina NextSeq	MiGS
**V06**	P1-2CT-D	*Chryseobacterium camelliae*	100	SAMN37538693	4097534	4100000	0.999	Illumina NextSeq	MiGS
**B09**	L1-17CB-X	*Enterobacter*	100	SAMN37538671	4906083	4900000	1.001	Illumina NovaSeq	LaVieBio
**Q13**	L1-19CT-E	*Exiguobacterium indicum*<br>*Exiguobacterium acetylicum*	52<br>48	SAMN37538682	3301099	3000000	1.100	Illumina NextSeq	MiGS
**7AKAJ03**	L1-8CT-P1-Y	*Klebsiella aerogenes*	100	SAMN37538670	5277583	5280000	1.000	Illumina NovaSeq	LaVieBio
**L21**	L1-4CT-C	*Klebsiella aerogenes*	101	SAMN37538677	5280467	5280000	1.000	Illumina NextSeq	MiGS
**M15**	P1-4CT-G	*Klebsiella aerogenes*	100	SAMN37538679	5277331	5280000	0.999	Illumina NovaSeq	LaVieBio
**P38**	P1-11CB-C	*Klebsiella aerogenes*	100	SAMN37538681	5083689	5280000	0.963	Illumina NextSeq	MiGS
**Q27**	P1-19CT-C	*Klebsiella variicola*<br>*Klebsiella pneumoniae*	54<br>46	SAMN37538685	5731213	5600000	1.023	Illumina NextSeq	MiGS
**Q26**	P1-19CT-D	*Kosakonia cowanii*	100	SAMN37538684	4814644	4800000	1.003	Illumina NextSeq	MiGS
**V50**	P1-2CT-F	*Lactococcus lactis*	100	SAMN37538694	2432953	2400000	1.014	Illumina NovaSeq	LaVieBio
**S39**	P1-12CT-C	*Leclercia adecarboxylata*	100	SAMN37538688	4844296	4800000	1.009	Illumina NovaSeq	LaVieBio
**T23**	P1-12CT-D	*Leclercia adecarboxylata*	100	SAMN37538690	4844236	4800000	1.009	Illumina NovaSeq	LaVieBio
**C19**	L1-3CT-T	*Microbacterium testaceum*	100	SAMN37538672	3477846	3900000	0.892	Illumina NextSeq	MiGS
**H13**	L1-16CB-P	*Paenibacillus glucanolyticus*<br>unclassified *Pseudomonas*<br>*Bacillus cohnii*	79<br>14<br>7	SAMN37538674	7634816	7000000	1.091	Illumina NextSeq	MiGS
**E04**	L1-5CB-Z	*Pantoea agglomerans*<br>*Pantoea vagans*	90<br>10	SAMN37538673	4789057	4800000	0.998	Illumina NovaSeq	LaVieBio
**L19**	P1-4CT-C	*Pantoea ananatis*	100	SAMN37538676	4941821	4600000	1.074	Illumina NovaSeq	LaVieBio
**L72**	P1-1CB-F	*Pantoea ananatis*	100	SAMN37538678	4974524	4600000	1.081	Illumina NovaSeq	LaVieBio
**J11**	L1-13CB-Z	*Pantoea vagans*<br>*Pantoea anthophila*	99<br>1	SAMN37538675	4894223	4890000	1.001	Illumina NextSeq	MiGS
**V04**	P1-12CT-B	*Pantoea vagans*<br>unclassified *Pantoea*	68<br>32	SAMN37538692	4778152	4890000	0.977	Illumina NextSeq	MiGS
**R67**	L1-11CT-E	*Pseudomonas parafulva*	100	SAMN37538687	4829812	4900000	0.986	Illumina NextSeq	MiGS
**U39**	L1-5CT-A	*Rahnella aquatilis*	100	SAMN37538691	5442646	5500000	0.990	Illumina NextSeq	MiGS
**M31**	P1-4CT-I	*Stenotrophomonas pavanii*<br>*Stenotrophomonas maltophilia*	97<br>3	SAMN37538680	4467315	4300000	1.039	Illumina NextSeq	MiGS
**S43**	P1-6CB-D	*Tatumella*	100	SAMN37538689	3202805	3300000	0.971	Illumina NextSeq	MiGS

Figure S1. Whole-genome details. Genome information for the fertilization-stage silk bacterial isolates used in this study, including whole genome sequencing results (including relative abundance for the assignment of taxonomy), NCBI GenBank accession number, the genome length (number of bases sequenced), the expected genome size based on the taxonomy prediction, the calculated genome coverage (genome length divided by expected genome size), the Illumina system, and the company that conducted the sequencing. MiGS provided bioinformatic analyses for all isolates.

Supplementary Text S1. In vitro testing of microbial traits

S1.1. Indole containing compound (auxin) production

The indole compound production assay (proxy for indoleacetic acid, IAA) was modified from Johnston-Monje & Raizada [6] which was based on Bric et al. [39]. Isolates were inoculated with 2 μL of a 3 mL LB overnight liquid culture onto 50 mL R2A agar plates amended with L-tryptophan (in ddH_2O: R2A powder 18.12 g/L, adjusted to pH 7.2 with NaOH and autoclaved, amended with 5 mM filter-sterilized L-tryptophan in ddH_2O before pouring plates) and incubated at 30 °C for 3 d. After an intermediate step of application of a nylon membrane and overnight refrigeration, a nitrocellulose membrane (BioTrace™ NT, Pure Nitrocellulose Blotting Membrane, P/N 66485, Pall Corporation, Pensacola, Florida, USA) was placed over the plate, which was parafilmed and left overnight at 4 °C. A new filter paper (Whatman filter paper no. 2, Cat. No. 1002-150, GE Healthcare Life Sciences, USA) was soaked with Salkowski reagent (0.01 M $FeCl_3$ in 35% $HClO_4$) for 30 min, and then the nitrocellulose membrane was transferred onto the filter paper. IAA production was indicated by a dark pink color change.

S1.2. Growth on low nitrogen media

Bacteria were tested for their ability to grow on media without added nitrogen as adapted from the American Type Culture Collection's "ATCC medium: 1312 *Azospirillum amazonense* (LGI medium)" and

contained molybdenum to facilitate biological nitrogen fixation. Specifically, 1-2 d old liquid cultures were grown in 3 mL LB and then loops used to streak isolates onto 15 mL ATCC LGI nitrogen-free agar plates (100 mm x 15 mm). The LGI media recipe was as follows: in ddH_2O: 0.2 g/L K_2HPO_4, 0.6 g/L KH_2PO_4, 0.02 g/L $CaCl_2•2H_2O$, 0.2 g/L $MgSO_4•7H_2O$, 0.002 g/L $Na_2MoO_4•2H_2O$, 0.01 g/L $FeCl_3$, 5 mL/L Bromothymol blue ($C_{27}H_{28}Br_2O_5S$, 0.5% in 0.2 N KOH), 5 g/L sucrose, 15 g/L Bacto-agar, prepared in glassware pre-washed with 4 M HCl, adjusted to pH 6.0 with HCl. The plates were incubated at 30 °C in an anaerobic chamber (Baker-Ruskinn Concept 500, gas mix: Linde, 10% H_2, 10% CO_2, 80% N_2) for 5 d. Isolates which grew after 5 d were restreaked onto pre-reduced 25 mL ATCC LGI nitrogen-free plates in the anaerobic chamber and incubated at 37 °C for 5 d. Candidates were considered positive if they grew on both the first and second set of restreak plates, in 3/3 replicates.

S1.3. PEG (desiccation) tolerance

This poly(ethylene glycol) tolerance protocol, a proxy for drought tolerance, was adapted from Hernández-Fernández et al. [40] and Latif et al. [41]. Isolates were grown for 1-2 d old as LB liquid cultures, then aliquots of 10 µL were inoculated in 500 µL LB (pH 7.2) amended with 0%, 10%, 30%, and 40% PEG-6000 on triplicate 96 deep well plates. Negative controls received no bacteria for background contamination. Isolates were randomized within a PEG-6000 concentration, sealed with a breathable membrane, and incubated at 30°C at 200 rpm for 2 d. OD_{600} readings were taken using a SpectraMax 384 Plus spectrophotometer (Molecular Devices, USA) using 110 µL aliquots, with fresh, sterile LB broth as the blank. Optical density measurements were normalized to the respective 0% LB OD_{600} reading and then multiplied by 100 to obtain a percentage, and then the mean percentage was calculated and categorized as: susceptible to PEG (0-8%), mild resistance (8-12%), moderate resistance (12-31%), moderate-high resistance (31-50%), or high resistance (>50%).

S1.4. Acid and aluminum tolerance

This acid and aluminum tolerance protocol was adapted from Huang et al. [42] and Lim et al. [43]. Bacteria were primed in sterile 96 deep well plates containing 500 µL per well of 50 µM $AlCl_3•6H_2O$ at pH 3.5 in glucose-$MgSO_4$ (GM) broth, which were then incubated at 30°C and 200 rpm in triplicate overnight. From these cultures, 10 µl per isolate were used to inoculate 0.1 mmol $AlCl_3•6H_2O$ at pH 3.5 in GM broth, which were again incubated at 30°C and 200 rpm in triplicate overnight. GM broth consisted of: 1% glucose, 0.05% peptone, 0.02% yeast extract, 0.02% $MgSO_4•7H_2O$, pH adjusted to 3.5 with HCl, followed by autoclaving, and then amended with filter-sterilized $AlCl_3•6H_2O$ (4 mM stock in cooled GM broth) to the concentration noted. Positive controls were the isolates inoculated into GM broth with no aluminum, while negative controls received no bacteria. The bacteria that survived the priming steps (OD_{600} >0.003 in all of the three replicates) were tested in 500 µL GM broth with 0%, 0.4 mM, 2 mM, and 4 mM $AlCl_3•6H_2O$ in GM broth; the inoculum used was from the last priming step using the middle OD_{600} read sample with a few exceptions. These test samples were again incubated at 30°C and 200 rpm in triplicate overnight. At the test stage, the samples on each of the triplicate plates were uniquely randomized, with the same pattern used across Al^{3+} treatments within a plate. OD_{600} readings were taken using 110 µL aliquots of each sample, in a SpectraMax 384 Plus spectrophotometer (Molecular Devices, USA), with fresh GM broth as the blank.

Supplementary Text S2. Details for whole-genome sequencing, gene annotation, and mining

S2.1. Whole-genome sequencing and gene annotation

Single colony liquid cultures of 25 isolates (including representatives from the most prevalent species and OTUs cultured, and some special inclusions) were used for bacterial genomic DNA isolation using the DNeasy UltraClean microbial kit (product number 1222450; Qiagen). Illumina sequencing was conducted by third parties (Lavie Bio Ltd and MiGS). At Lavie Bio Ltd. (Rehovot, Israel), libraries were generated using the TruSeq DNA Nano library preparation kit (KAPA HyperPrep kit, # KK8504), and sequencing was undertaken using the NovaSeq 6000 platform. The EvoCAT (Evogene Clustering and

Assembly Toolbox) pipeline was used to filter raw reads (Phred quality score of 30) followed by *de novo* assembly. At MiGS (Pittsburgh, USA) the NextSeq 2000 platform was used. Quality control and adapter trimming were performed using bcl2fastq (version 2.20.0.445, default parameters) [44]. Both Lavie Bio and MiGS sequences were bioinformatically analyzed at MiGS. Species-level taxonomy was undertaken using MetaPhlAn3 (Tool: 3.0.7, December 9, 2020, database version; default parameters + 'add_viruses) [45]. The short read mode of Unicycler (version 0.4.8 with default parameters) [46] was used as a SPAdes [47] optimizer to create short-read assemblies. Gene annotation was undertaken using PGAP software (Tool: Build5132, database version January 11, 2021, with default parameters) [48] and prokka (tool and database version: 1.14.5; using default parameters + '—rnammer' + '—rfam', added '—metagenome' when processing metagenomic/unclassified samples, added '—kingdom Viruses' when processing viral and bacteriophage samples) [49].

S2.2. Whole-genome mining

The gene annotation files for the isolates were combined into one Excel file. Genes of interest were searched for (Ctrl-F) using multiple variations of the names, short forms, acronyms, and related search terms. Applicable genes were copied into a new spreadsheet and the number of occurrences of the genes was tallied. To confirm genes were identified/annotated correctly, an amino acid sequence for a representative from each unique gene identifier was searched using the protein-protein Basic Local Alignment Search Tool (BLASTp) and annotations were compared to the best matches in the non-redundant protein sequences database at NCBI.
